# Top-down influence on gaze patterns in the presence of social features

**DOI:** 10.1371/journal.pone.0183799

**Published:** 2017-08-24

**Authors:** Aleya Felicia Flechsenhar, Matthias Gamer

**Affiliations:** Department of Psychology, Julius Maximilian University of Wuerzburg, Wuerzburg, Germany; University of Verona, ITALY

## Abstract

Visual saliency maps reflecting locations that stand out from the background in terms of their low-level physical features have proven to be very useful for empirical research on attentional exploration and reliably predict gaze behavior. In the present study we tested these predictions for socially relevant stimuli occurring in naturalistic scenes using eye tracking. We hypothesized that social features (i.e. human faces or bodies) would be processed preferentially over non-social features (i.e. objects, animals) regardless of their low-level saliency. To challenge this notion, we included three tasks that deliberately addressed non-social attributes. In agreement with our hypothesis, social information, especially heads, was preferentially attended compared to highly salient image regions across all tasks. Social information was never required to solve a task but was regarded nevertheless. More so, after completing the task requirements, viewing behavior reverted back to that of free-viewing with heavy prioritization of social features. Additionally, initial eye movements reflecting potentially automatic shifts of attention, were predominantly directed towards heads irrespective of top-down task demands. On these grounds, we suggest that social stimuli may provide exclusive access to the priority map, enabling social attention to override reflexive and controlled attentional processes. Furthermore, our results challenge the generalizability of saliency-based attention models.

## Introduction

According to traditional models, visual attention is guided by both cognitive endogenous (top-down) factors, such as knowledge, expectation and current goals, and exogenous (bottom-up) factors that reflect sensory stimulation. This insight is based on numerous experimental laboratory paradigms using simple stimuli to disentangle and identify mechanisms underlying attention control (for reviews see [1,2]. However, the conclusions of these setups are not necessarily transferable to the real world, where sensory signals continuously compete for the brain’s limited processing resources and stimuli and responses are inextricably linked. As such, prominent aspects in our environment are not only important due to their features, but also through their behavioral relevance. In turn, distinctive sensory stimuli attract attention more effectively when they are relevant or contingent to the task at hand [3].

A large body of research that was devoted to predicting gaze behavior, relied on the calculation of so-called saliency maps, which filter early features, such as orientation, contrast intensity and color. For example, the prominent, graph-based saliency (GBVS) algorithm by Harel, Koch & Perona [4] acts by decomposing the image into a series of feature maps and generating activation maps on certain feature channels based on graph theory. These maps are then combined into a single saliency map revealing locations that stand out in terms of their low-level features from the background. Accordingly, attention should be allocated to locations in the scene depending on the saliency in the computed map using a winner-takes-it-all mechanism. These maps have become an integral component of many subsequent models of gaze allocation, suggesting a correlation between low-level features in scenes and fixation selection by humans (for reviews see [5,6]). However, they do not necessarily drive attention causally, but contingent on higher-order statistics [7] as visual saliency seems to provide a poor account of eye fixation patterns in complex visual scenes [8,9] and lacks prediction of overt spatial orienting for long exposure [10], where intrinsic and strategic aspects are thought to be prominent. Other studies concentrating on social aspects of visual attention, have shown that saliency models were also inaccurate in describing fixation selection for socially relevant stimuli, such as human faces [11], as well as scenes with whole bodies [12–14], showing a gaze behavior that was uncorrelated to low-level image statistics even from the first saccade onwards. These findings indicate that social stimuli may engage special perceptual processing and provide exclusive access to the priority map, enabling social attention to override reflexive and controlled attentional processes. To explicitly disentangle to what degree this social override occurs, we conducted an eye tracking study introducing top-down demands in form of tasks with different complexity to non-social aspects of the stimuli, rendering the social aspects to be uninformative for the viewer. Most findings concerning preferential viewing of social information were conducted under free-viewing conditions or have introduced tasks encouraging fixations onto social information (e.g. [14,15]). To our knowledge, the influence of varying degrees of top-down demands that explicitly require the direction of attention away from the social content has not yet been investigated. Early studies by Yarbus [16] already suggest that the selection of gaze information may depend on the task that is assigned to participants and the social content of the scene. A replication study of DeAngelus and Pelz [17] using modern eye tracking, confirmed these results, revealing different scanpath patterns for distinct tasks. We hypothesized that attention will be devoted to social scene elements due to their behavioral relevance for human beings even when they do not need to be scanned to accomplish the experimental task and this preference may already be evident in very early fixations.

## Materials and methods

### Participants

The study was approved by the ethics committee of the German Psychological Society (DGPs) and conducted according to the principles expressed by the Declaration of Helsinki. All participants provided written informed consent and received payment for their participation. Forty five subjects voluntarily took part in the experiment. Five were excluded due to missing responses in more than 25% of all trials or low eye tracking data quality (>20% missing baseline values or baseline outliers, see below), respectively. This sample size allowed us to detect medium effect sizes (Cohen’s *f* = 0.25) for fixation differences between the four experimental tasks (see below) with a power of at least 95% when assuming a correlation of *r* = .50 between factor levels.

The age of the final set of participants (21 women) ranged from 18–35 years (*M* = 24.45 years, *SD* = 4.15 years) and consisted of 31 students from various disciplines, as well as 9 employed subjects. All participants had normal or corrected-to-normal vision with the option of wearing contact lenses. Women were asked to refrain from using eye make-up. Three participants were left-handed (all women). The average amount of school years was no lower than a General Certificate of Secondary Education with a minimum duration of 10 school years (*M* = 12.33 years, *SD* = 0.92 years) and did not differ between men and women (*t*_(38)_ = 1.04, *p* = .304).

### Design

A total of 120 naturalistic stimuli showing negative, neutral and positive scenes, were used for the experiment. Half of them included human beings (referred to as “social images” in the current article) and the other half did not depict human beings, but showed landscapes, objects, animals, etc. (referred to as “non-social images” here). Four different tasks were included with tasks 2–4 specifically addressing non-social attributes: (1) free viewing condition, in which participants were allowed to look wherever they pleased, (2) definition, which entailed naming the color of a four-wheeled vehicle in the picture (e.g. a car, a bus, a truck), (3) counting, which required determining the number of blue objects in the picture and 4) estimation, where the percentage of white in the picture had to be estimated. We decided to use these additional three different tasks instead of merely a single one to examine potential influences of complexity on social attentional mechanisms. Furthermore, we chose tasks that also aimed at global (estimation) as compared to local (definition, counting) scanning to investigate differences herein. Therefore, harder tasks requiring higher top-down employment may interfere more with attention towards social features than easier tasks and tasks demanding local gaze distributions may affect social attention more than those encouraging global scanning. One of these tasks had to be completed during the total presentation time of 10s for every stimulus. Participants were explicitly told that the blue objects they had to count were non-social and not part of human beings (e.g., no clothes). Notably, not all objects within a stimulus were always uniform, but were chosen as such that they could be clearly classified as individual objects. Also, no vehicle was ever occupied to ensure the non-social aspect of the task. Furthermore, stimuli were chosen as such that the number of humans depicted in the scene varied, as well as the aspect of high saliency of faces and bodies. Moreover, depicted subjects were distributed across the whole scene to reduce a central bias and their size varied. All stimuli were applicable to all tasks (with the exception of the color definition tasks, which was limited to 36 pictures) and randomized for each task and subject to avoid stimulus-specific effects. Thus, in total, each subject accomplished each task for 15 social and 15 non-social images, respectively.

### Stimuli and tasks

Stimuli were presented on a 24” LG 24MB65PY-B screen (516.9 x 323.1 mm) with a resolution of 1920 x 1200 pixels and a refresh rate of 60 Hz. The viewing distance was 50 cm for each participant to view stimuli of 1200 x 900 pixels, resulting in a visual angle of 35.81° x 27.24°. The stimuli showed real-life situations that were either photographed or chosen from the internet (e.g. Google picture search, Flickr) and had to fulfill the requirements of all four tasks to be used randomly for different participants. Some pictures were modified to avoid writings or labels and adjusted in luminance and contrast to better match the rest of the stimulus set. Further, their resolution had to be sufficient in quality to rescale them accordingly. Image editing was performed with the software GIMP (version 2.8.16; GNU Image Manipulation Program, The GIMP Team).

Each trial began with the written command (black letters on a uniform grey background) defining the task for the subsequent stimulus, shown for 2s. This was followed by a fixation cross lasting for 2s. Afterwards, the stimulus was presented for a total of 10s, during which the participant had to gather an answer to the task and indicate this by clicking the left mouse button. This was used as a reaction time measure. Even after the click, the stimulus would stay on screen until the 10s passed. During this time participants were free to look wherever they pleased. At stimulus offset, an answer scale would appear on screen, requiring participants to choose one of eleven possible options for each task (defining: color palette, counting: from ≤ 5 to ≥ 15 in steps of 1; estimating: between 0% and 100% in steps of 10%) with the exception of the free-viewing condition, which depicted an OK button that had to be clicked to continue with the next trial. Inter-trial-intervals (ITI) amounted to 3-7s randomly drawn from a uniform distribution. A uniform grey screen was shown during the ITI. Eye tracking data were recorded during the tasks with a sampling rate of 1000 Hz using a tower mounted EyeLink 1000 plus system (SR Research Ltd., Ottawa, Canada) with a 25mm lens.

### Procedure

Upon arrival to the laboratory, participants were informed about the experiment, were asked to sign the consent form and then completed a short questionnaire concerning sociodemographic data (age, sex, profession and handedness). Afterwards, instructions concerning the tasks were given verbally and by means of printed examples, as well as for the eye tracking. To become acquainted with the procedure, 8 training trials were conducted with a separate set of pictures including every task twice. The fixation cross was to be fixated during the whole duration of its presence. During stimulus presentation, reading of task instructions, times for which the screen was blank or the time after their response, participants should feel free to change their gaze and look wherever they pleased. However, blinks during the recording time of the stimulus presentation should be avoided. Starting the experiment, the eye tracking system was calibrated using nine points and subsequently validated, followed by the actual task. Stimulus and task order was randomized across participants and the experiment was divided into four blocks to ensure small breaks in between and opportunities to rest the eyes. After completion of the experiment, participants were asked to rate the perceived difficulty of the tasks they had completed.

The experiment was programmed with the Experiment Builder Software (version 1.10.1630; SR Research Ltd., Ottawa, Canada) and data processing and analysis was performed using the open-source statistical programming language R (www.r-project.org) and Matlab® R2011b (Mathworks, Inc., Natick, MA, USA). A univariate analysis approach as implemented in the *ez* package (version 4.3; [18]) was used for all repeated-measures analyses of variance (ANOVAs). The a-priori significance level was set to α = .05 for all statistical tests and general ɳ^2^ is reported as an effect size index. Huynd-Feldt’s ε is reported for all repeated-measures ANOVAs containing more than one degree of freedom in the numerator to account for potential violations of the sphericity assumption. Cohen’s *d* is reported as an effect size estimate for two-sample and paired *t*-tests [19].

### Data processing and analysis

#### Behavioral data

Reaction times were calculated as the difference between stimulus onset and the first mouse click during stimulus presentation. If no click was given, the reaction time was set to the total presentation time of 10s. Differences in reaction times were analyzed as a function of stimulus content (social vs. non-social) and task (defining, counting, estimating) using a 2 x 3 repeated-measures ANOVA. Complexity of each task was rated by participants after completion of the experiment on a scale of 1 (very easy) to 6 (very hard). Differences in difficulty ratings were analyzed in a one-way ANOVA with the factor task.

#### Eye tracking data

For the analysis of the eye-tracking data, we first calculated visual saliency maps. A large number of different algorithms have been suggested for such purpose [5]. We decided to primarily rely on the graph-based visual saliency algorithm (GBVS) by Harel, Koch & Perona [4]. It uses graph algorithms for saliency computations, forming activation maps on certain feature channels and then normalizing them to highlight conspicuity, admitting combinations with other maps. The GBVS algorithm is available as Matlab® source code, it is applicable without initial training and it performs well in predicting fixations in free viewing conditions [20,21]. It is important to note that the currently reported results do not strictly depend on this saliency algorithm since similar results were obtained for the algorithm by Itti and colleagues [22]. The GBVS algorithm generates maps that range between 0 to 1 and depict the distribution of visual saliency across the image. The current set of social and non-social scenes was comparable regarding mean saliency (*t*_(118)_ = 0.086, *p* = .93, *d* = .016, average mean for social scenes: .25, average mean for non-social scenes: .25) and saliency variation (*t*_(118)_ = 0.031, *p* = .98, *d* = .006; average *SD* for social scenes: .20, average *SD* for non-social scenes: .20). Saliency maps were used for determining image regions with higher as compared to lower visual saliency (see below).

In a second step, eye movements were segmented into saccades and fixations using velocity and acceleration thresholds of 30°/s and 8000°/s^2^, respectively, for saccade detection. Time intervals between saccades were defined as fixations. For all eye movement measures, fixations were drift corrected with reference to a baseline period of 300 ms before stimulus onset (i.e. when the central cross was fixated). Outliers of baseline coordinates were identified using a recursive outlier removal procedure that was applied separately to x- and y-baseline-coordinates. For each participant the highest and lowest baseline coordinates were temporarily removed and the mean and standard deviation were calculated for the remaining data. If either of the two values fell outside an interval bounded by 3 standard deviations from the mean, it was removed completely. If the data points fell within the interval, they were returned to the data set. This procedure was continued until no more data points were discarded. Trials with invalid baseline position data were replaced by the means of all valid baseline positions, including a removed x or y baseline coordinate or missing baseline data (proportion of social and non-social scene trials: *M* = 5.54%, *SD* = 5.68% and *M* = 4.96%, *SD* = 4.44%, respectively). For further analyses, trials containing too many blinks were excluded (trials with a blink-free time period of less than 80% of the whole trial: *M* = 13.0%, *SD* = 0.07%) and fixations were drift corrected and then used to create fixation density maps. The first fixation was not considered since it usually overlapped from the fixation period before stimulus onset. An empty two-dimensional map (1200 x 900 pixels) was generated for each participant and stimulus. The respective fixations were weighted by their fixation durations in milliseconds, which were added at the pixel position of the fixation. The resulting map was then smoothed with a two-dimensional isotropic Gaussian kernel with a standard deviation of 36 pixels or 1° of visual angle using the R package *spatstat* (version 1.45.0; [23]). The total smoothing kernel amounted to 2° of visual angle (one standard deviation in positive and one in negative direction) to resemble the functional field of the human fovea centralis. The fixation maps were then normalized to range from 0 to 1. Eye movements up to the point of the mouse click were analyzed, to investigate task-relevant fixations, as well as the period after the mouse click to examine potential changes in viewing behavior after completion of the task.

**Fig 1:**
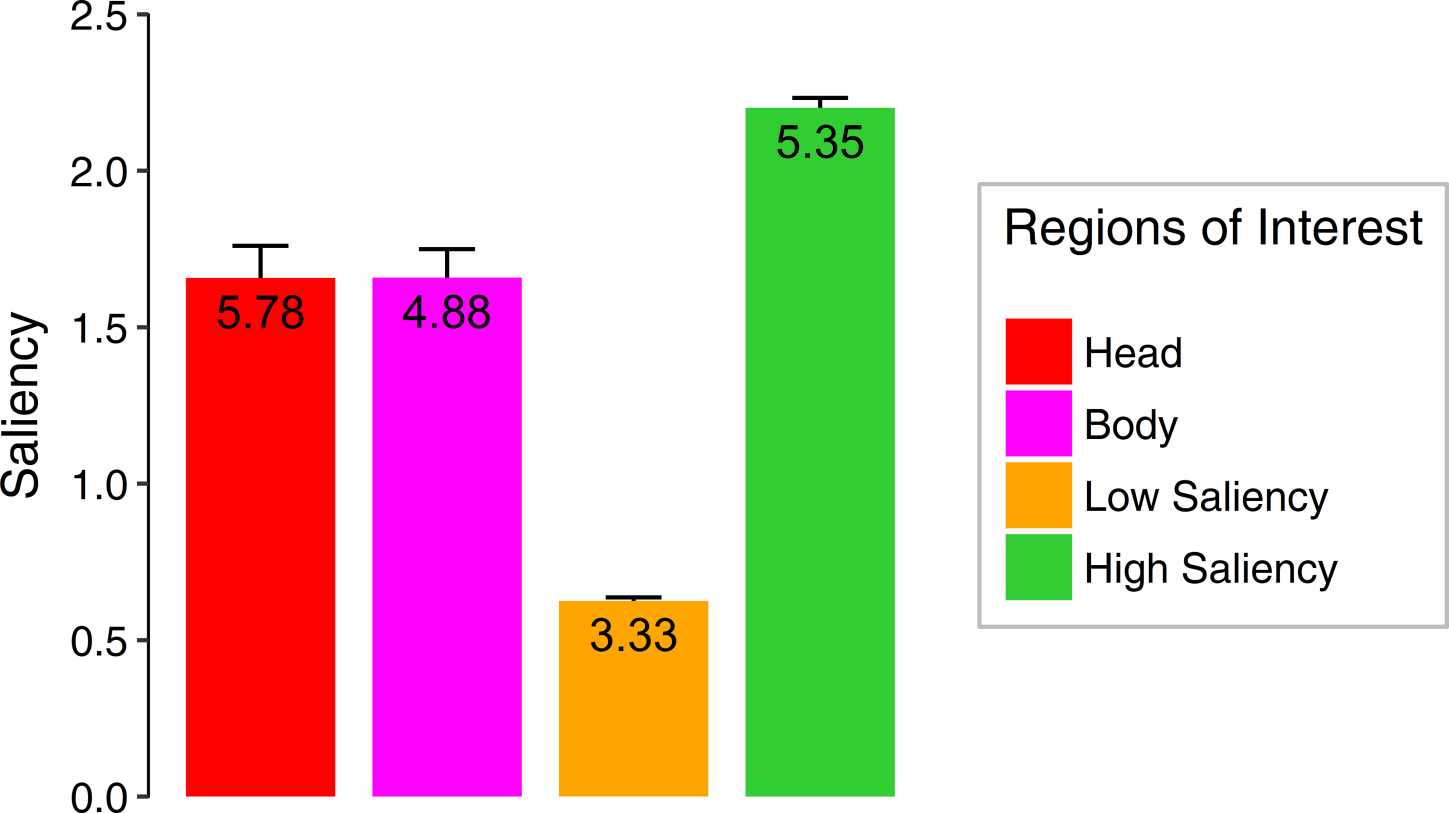
The graph depicts the mean saliency values for the different regions of interest (head, body, areas of low and high saliency) for all social images according to the graph-based visual saliency algorithm [4]. The values depicted within the bars describe the mean number of ROIs per category and image across all social stimuli. Error bars represent standard errors of the mean.

In a third step, we introduced regions of interest (ROIs) to investigate the distribution of fixations onto the social and non-social features of the stimuli. Specifically, pixel coordinates were defined for head, body and areas with lower and higher saliency to all stimuli containing social information (*n* = 60) and areas with lower and higher saliency to all non-social stimuli (*n* = 60). Head and body ROIs were manually drawn in GIMP and each ROI pixel was assigned a certain color. In order to define the ROIs for saliency, the saliency maps for social scenes were considered for those image regions, which had not already been assigned to the head or body ROI, while the whole scene was considered for non-social stimuli. Saliency values smaller or equal to the eighth percentile of the saliency distribution were defined as areas of lower saliency. Although the criterion was arbitrary, this cut-off allowed identification of image regions which were highly salient, but contained no social information, which deemed essential to disentangle potential effects of attentional allocation to social versus physically salient information. To ensure that social features did not represent the most salient image regions, we calculated the mean saliency of each ROI for each social scene and divided the resulting values by the mean saliency of the whole scene. Further, we calculated a one-way ANOVA with the factor ROI to confirm that social features were less salient than areas of high saliency (main effect of ROI: *F*_(3,117)_ = 113.18, ε = .53, *p* < .001, η^2^ = .52) (Fig 1). Additionally, we determined the number of ROIs per category (head, body, low saliency, high saliency) per image across all social images. These values are also displayed in Fig 1. Importantly, the numbers of the most relevant social ROI (i.e., heads, *M* = 5.78, *SD* = 5.95) and non-social ROI (i.e., highly salient regions, *M* = 5.35, *SD* = 3.60) did not differ significantly (*t*_(59)_ = 0.59, *p* = .56, *d* = .004).

**Fig 2:**
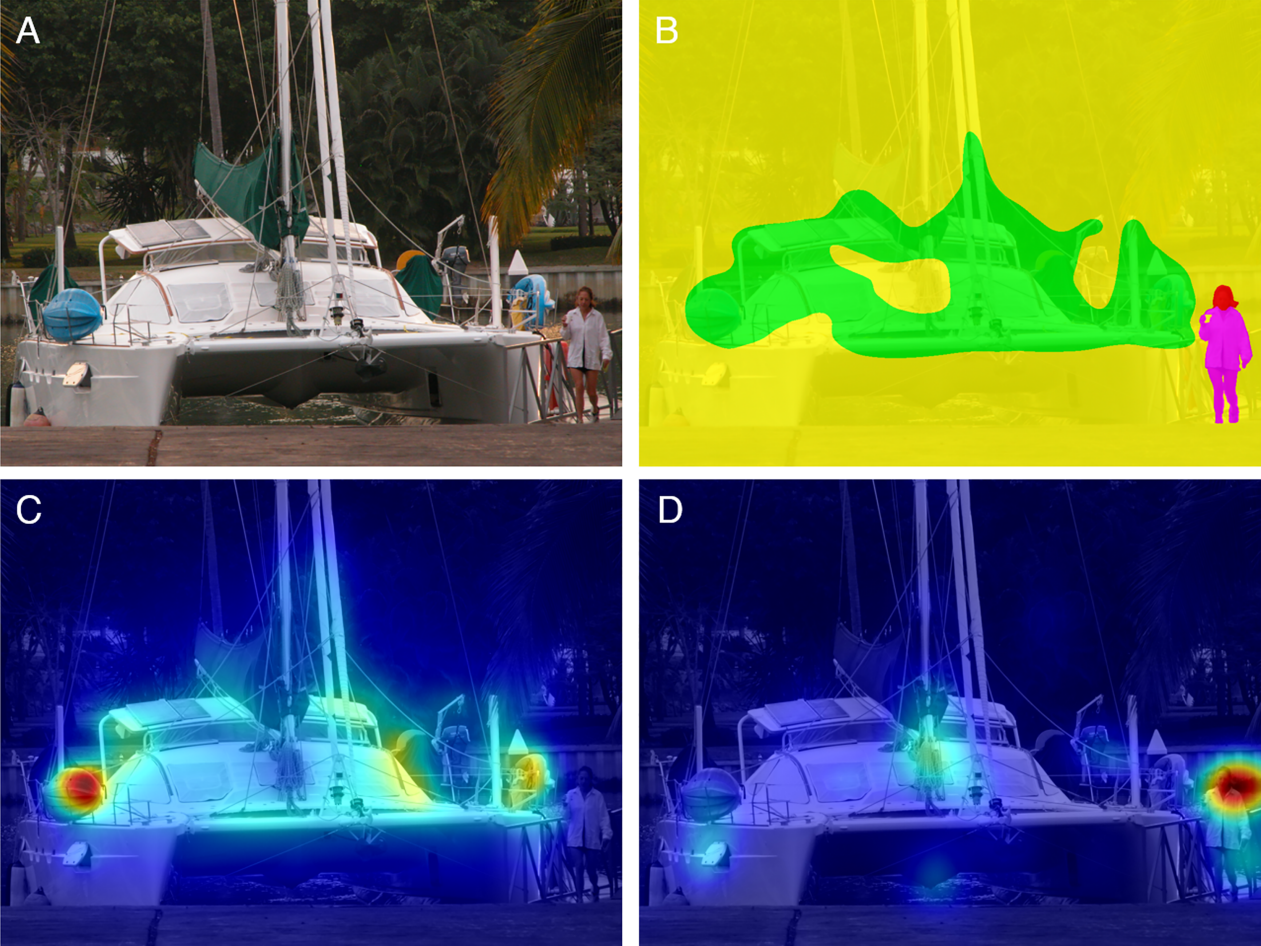
Example of a social stimulus as published in End & Gamer (2017) [13]. (A) Original scene. (B) Example of defined regions of interest for head (red), body (magenta), low saliency (yellow) and high saliency (green). (C) Overlay of a saliency map according to the Graph-Based Visual Saliency algorithm [4] with cool colors representing low salient regions and warm colors defining areas of high saliency. (D) Overlay of a fixation density map derived from fixation patterns of 31 participants who viewed the stimulus for 10s under free-viewing conditions. Image taken with permission from the Nencki Affective Picture System [24]. Please note that the stimulus shown here was not used in the current study and is only depicted to illustrate the current data analysis strategy.

The defined ROIs and the fixation density maps were used to determine the relative extent to which each ROI was fixated. For this, the sum of fixation density values was calculated for each ROI and then divided by the sum of fixation density values for the whole scene. An example stimulus with respective ROIs, saliency and fixation density maps is depicted in Fig 2 (these images were adapted from End & Gamer (2017) whose analysis was followed closely in this study [13]). The proportion score was then normalized by taking the size of the ROI into account to control for increased fixations onto larger rather than smaller areas (see also [12]). The mean for this relative area-normalized sum of fixation density was calculated for each ROI across all social scenes for each participant as a function of time window (i.e., before acknowledging task completion and after the mouse click). To analyze fixation densities as a function of the experimental manipulations, several ANOVAs were calculated: A 4 x 4 repeated-measures ANOVA with the factors task (free-viewing, color definition, object counting and estimation) and ROI (head, body, areas of low saliency and high saliency) was run for the task-relevant time window (for the free-viewing condition, this amounted to the whole presentation duration). Task-relevant and task-irrelevant time windows were compared by a 2 x 3 x 4 repeated-measures ANOVA with factors time window, task (now excluding the free viewing task) and ROI. Similar analyses were conducted for the non-social stimuli, using a 4 x 2 repeated-measures ANOVA for the task-relevant time window with the factors task and ROI (low and high saliency) and a 2 x 3 x 2 repeated-measures ANOVA with the factors time window, task (again excluding the free viewing task) and ROI. Additionally, since we differentiated the background of each scene into areas of low and high saliency, we conducted a further analysis in which we compared areas of low and high saliency also within social ROIs using the same criteria for definition as for the background. Thus, head and body ROIs were dissociated into regions with low saliency (saliency values less or equal than the eighth percentile of the saliency distribution) and high saliency (remaining areas). Herewith, we could examine fixation densities across different regions in the scene (head, body, background) depending on the saliency distribution (low and high) for each task. This was accomplished using a 4 x 3 x 2 ANOVA with repeated measures using the factors task, regions and saliency.

Fourth, we carefully examined the initial fixation changes towards ROIs to reinforce our findings, but also to gain more insight into the processing speed and potential automatic attention-grabbing effect of social stimuli. For each participant, the relative frequency that each ROI was fixated across all social scenes was determined by dividing the frequency that each ROI was fixated by the frequency that any ROI was fixated for each of the first three fixations after stimulus onset. The relative frequency scores were normalized by considering the mean area of the respective ROI across all social scenes when represented in the according relative frequency score. Social scenes, were analyzed by a 4 x 4 x 3 repeated-measures ANOVA with the factors task, ROI and fixation number. To directly compare initial fixations between both social and non-social images, we conducted a 2 x 4 x 2 x 3 ANOVA incorporating the additional factor stimulus category and comparing ROIs for areas of low and high saliency (factors stimulus category, task, ROI and fixation).

It is of note that the trials of which baseline position data were replaced by mean baseline position data for drift correction were removed from the analyses of the first fixations, as participants may not have fixated the central cross directly before the onset of the scene in these trials. Hence, the starting position may differ between these trials as compared to trials in which the cross was fixated. The construction of fixation density maps would not be influenced by this disparity, since it comprises the fixations of the entire viewing duration of the scene. However, it may influence the locations of fixations occurring very early after the onset of a scene which would specifically effect analyses of the first three fixations.

## Results

### Behavioral data

**Fig 3:**
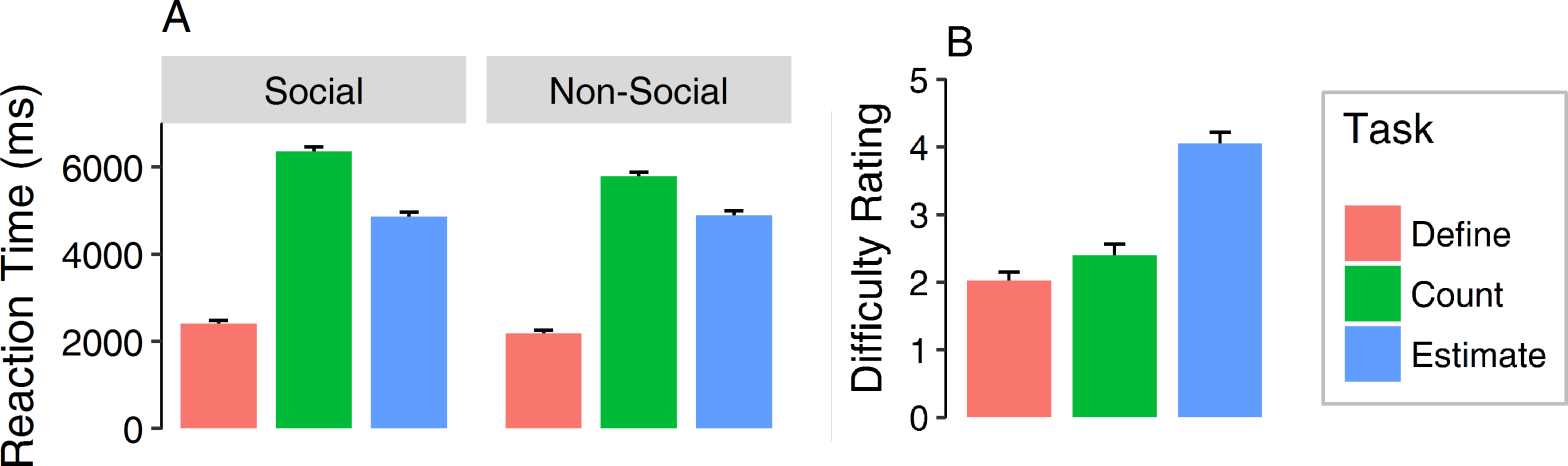
**Reaction times (ms) as a function of task and stimulus category (A) and difficulty ratings for the different tasks (B).** Error bars represent standard errors of the mean.

Reaction Times were longest for counting objects followed by estimating and color definition (Fig 3A) resulting in a main effect of task (*F*_(2,78)_ = 193.29, ε = .96, *p* < .001, η^2^ = .56). When comparing social and non-social stimuli, a main effect of stimulus category becomes apparent (*F*_(1,39)_ = 21.57, ε = 1, *p* < .001, η^2^ = .008), showing slightly higher reaction times for social stimuli. A two-way interaction between task and stimulus category (*F*_(2,78)_ = 8.17, ε = .99, *p* < .001, η^2^ = .008) depicts higher reaction times for object counting and color definition for social stimuli than non-social ones, whereas estimation tasks required slightly longer reaction times for non-social stimuli. Concerning the complexity ratings, estimating was generally rated the hardest and color definition the easiest resulting in a main effect of task (*F*_(2,78)_ = 55.02, ε = .99, *p* < .001, η^2^ = .46). This evaluation is somewhat reflected in the reaction time data, as the easiest definition task was also the one with the lowest response times. However, counting objects seemed to require a longer focus than estimation, even though estimation was rated the hardest (Fig 3B).

### Eye tracking

#### Task-relevant viewing behavior

**Fig 4:**
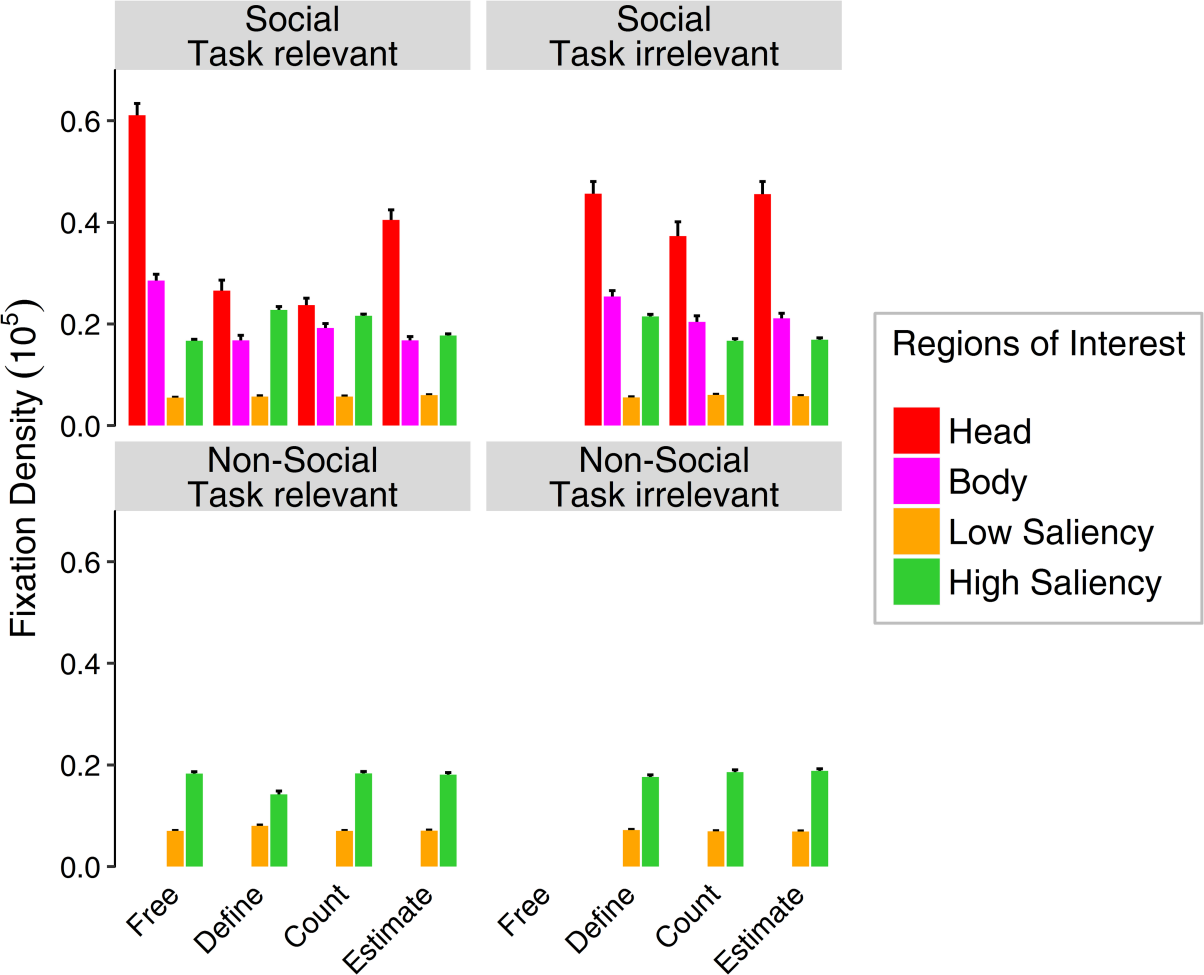
Direct comparison of relative area normed fixation density for social (above) and non-social stimuli (below) on different regions of interest for two different time windows (before and after a given response) as a function of task. The time window before the mouse click is referred to as “task relevant” (left panels), as it reflects possible top-down strategies on viewing behavior that were induced by the explicit tasks. The time window after completing the task is termed “task irrelevant” (right panels), as attentional allocation in this period should be less governed by task instructions. As there was no postulated task for the free-viewing condition, the task irrelevant time window does not contain any data for this modality. Viewing behavior for non-social stimuli was analyzed without social regions of interest, but allowed for comparing relative area normed fixation densities for areas of low and high saliency. Error bars indicate standard errors of the mean.

Analyses of the relative area-normed fixation density on social stimuli revealed a main effect of ROI (*F*_(3,117)_ = 302.51, ε = .46, *p* < .001, η^2^ = .67), showing a higher fixation densities on heads than on areas of high saliency, a main effect of task (*F*_(3,117)_ = 53.70, ε = 1, *p* < .001, η^2^ = .22), depicting higher fixation densities for free-viewing than for estimation, then color definition and lowest for object counting. Furthermore, we also found an interaction between ROI and task (*F*_(9,351)_ = 66.66, ε = .44, *p* < .001, η^2^ = .42) emphasizing that heads were fixated the longest in the free-viewing condition, followed by the estimation task, then the color definition and the smallest difference between heads and areas of high saliency was found in the counting task (Fig 4, left panel). Attention towards heads was most diminished in the easiest task, namely the color definition, but remained superior to image regions with high physical saliency (heads: *M* = 0.26, areas of high saliency: *M* = 0.23). Separate analyses were conducted for selection behavior in terms of fixation frequency as opposed to the reported analyses of fixation densities. Additionally, relative fixation densities were further analyzed as well, taking into account not only the area of the ROI, but also the number of ROIs within each category for each stimulus. Since both of these analyses provided highly similar results to the ones presented here, they are not described further.

#### Task-irrelevant viewing behavior

A comparison of the two time windows (before and after the participants’ response to the task) with the factor time confirmed these effects with an additional main effect of time (*F*_(1,39)_ = 37.19, ε = 1, *p* < .001, η^2^ = .04) with different fixation durations before and after the response to the given task. A main effect of task remained (*F*_(2,78)_ = 4.98, ε = 1, *p* = .009, η^2^ = .01), which largely resembles the free-viewing condition before the click with an additional main effect of ROI (*F*_(3,117)_ = 192.79, ε = .40, *p* < .001, η^2^ = .57) depicting a preference to focus on heads compared to other aspects of the picture. Furthermore, interactions between all three aspects was found: task and ROI (*F*_(6,234)_ = 13.21, ε = .45, *p* < .001, η^2^ = .059), task and time (*F*_(2,78)_ = 8.86, ε = .99, *p* < .001, η^2^ = .01), as well as ROI and time (*F*_(3,117)_ = 45.78, ε = .56, *p* < .001, η^2^ = .09), revealing higher fixation durations for heads as compared to other ROIs in the tasks of color definition and object counting after completion of the task compared to before the response. An additional three-way interaction (*F*_(6,234)_ = 8.09, ε = .51, *p* < .001, η^2^ = .03) confirms the tendency to prioritize social information when given the choice, even if a task preceded that required attention towards other aspects. In direct comparison, the attention towards heads is slightly diminished by the given task (before response), yet regains higher fixation durations after its completion (Fig 4).

#### Saliency effects within social regions of interest

**Fig 5:**
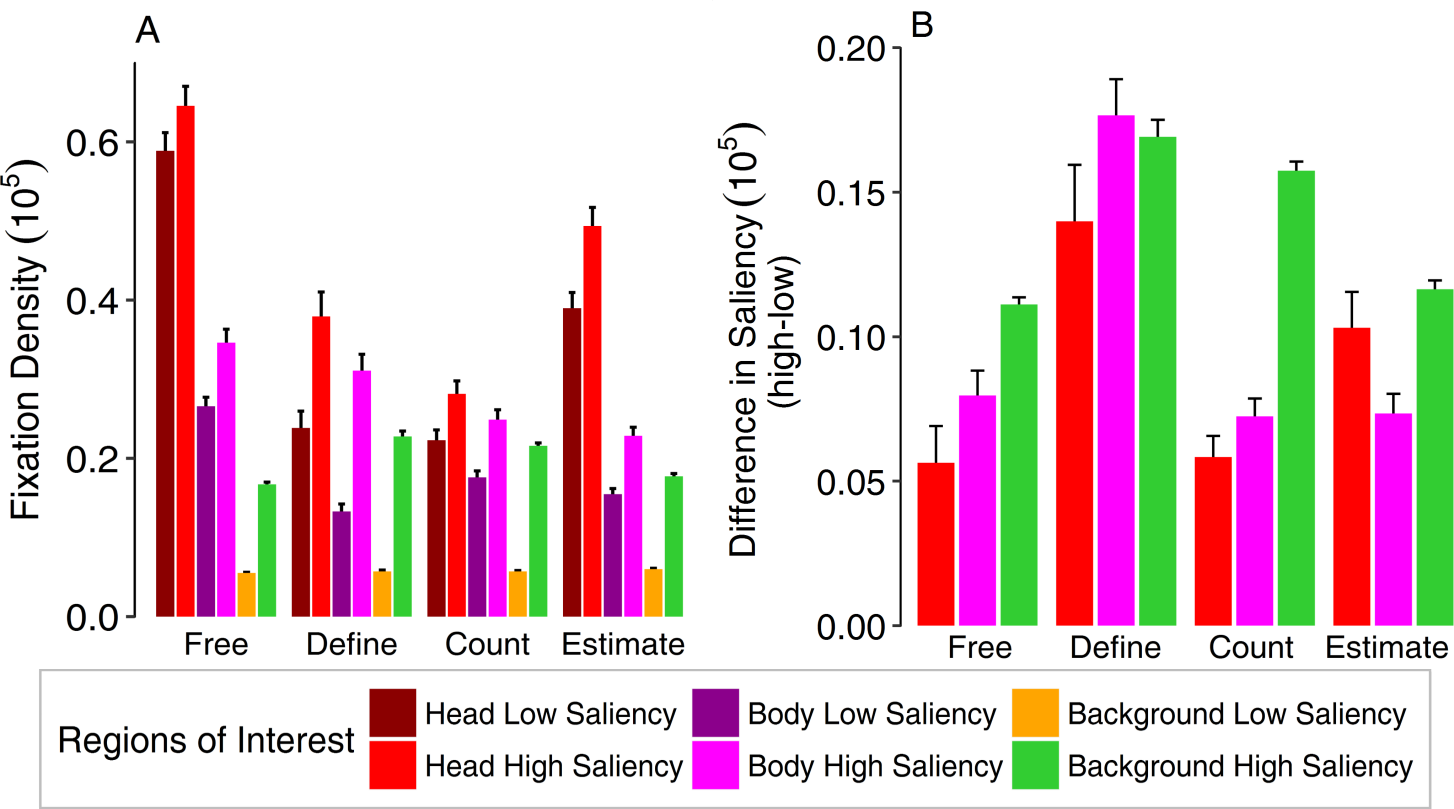
(**A**) Relative area normed fixation densities as a function of ROI (head, body and background) and saliency (high vs. low) of these regions across different tasks. (**B**) Difference in relative area normed fixation densities between highly salient and less salient areas for each region of interest (head, body and background). Please note that the color coding of ROIs was adapted to the values of the highly salient regions. Error bars represent standard errors of the mean.

The ANOVA taking into account the saliency distribution also within social ROIs, resulted in a main effect of salience (*F*_(1,39)_ = 611.98, ε = 1, *p* < .001, η^2^ = .20), indicating that participants rather fixated areas of high saliency as compared to areas of low saliency across all ROIs. This effect was qualified by an interaction of saliency and ROI (*F*_(2,78)_ = 22.16, ε = .97, *p* < .001, η^2^ = .01) (see Fig 5A), demonstrating larger effects of saliency on fixation densities for the background as compared to social ROIs. Furthermore, a main effect of task (*F*_(3,117)_ = 44.79, ε = .95, *p* < .001, η^2^ = .22) again described differences between tasks with generally higher fixation densities for free-viewing and estimating compared to defining and counting. A main effect of ROI was evident (*F*_(2,78)_ = 259.71, ε = .66, *p* < .001, η^2^ = .52), as head ROIs were still viewed for longer durations than other ROIs. Interactions between saliency and task (*F*_(3,117)_ = 9.50, ε = .83, *p* < .001, η^2^ = .01), as well as task and ROI (*F*_(6,234)_ = 55.87, ε = .59, *p* < .001, η^2^ = .28) were also significant but qualified by an additional three-way interaction of saliency, task and ROI (*F*_(6,234)_ = 3.31, ε = .71, *p* = .004, η^2^ = .006). These effects indicate different fixation densities onto highly salient and less salient regions depending on the content (i.e., social vs. non-social regions) as well as the task. To further illustrate the actual influence of saliency onto viewing behavior towards these regions, we calculated the difference between high and low salient regions of each ROI. These results showed that difference values for head and body ROIs were lower than those for the background for all conditions except the definition task (see Fig 5B). Hence, saliency generally played a larger role in drawing attention for non-social aspects than for social ones, especially heads.

#### First fixations

**Fig 6:**
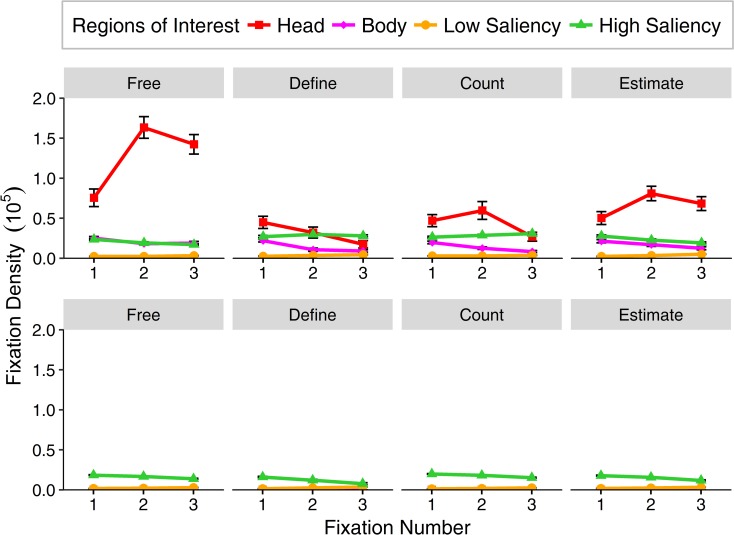
Relative area normed fixation density of the first three fixations after stimulus onset (before response) on the different regions of interest for all tasks separately. Top row: first fixations for social stimuli on four regions of interest (head, body, areas of low saliency and high saliency), bottom row: first fixations for non-social stimuli on two regions of interest (areas of low and high saliency). Error bars indicate standard errors of the mean.

The first three fixations were more closely analyzed with regard to the preference of specific ROIs (Fig 6). Over all tasks, a distinction of initial fixations on the head can be seen compared to the other areas resulting in a main effect of ROI (*F*_(3,111)_ = 163.55, ε = .36, *p* < .001, η^2^ = .41). Additionally, a main effect of task (*F*_(3,111)_ = 48.30, ε = .71, *p* < .001, η^2^ = .09) depicts slightly different viewing behavior for the different tasks, especially color definition and a significant interaction between ROI and task (*F*_(9,333)_ = 44.09, ε = .28, *p* < .001, η^2^ = .24) can be attributed to changes across tasks driving attention away from heads and onto aspects potentially relevant for the tasks. This is also underpinned by a main effect of fixation number (*F*_(2,74)_ = 14.61, ε = 1, *p* < .001, η^2^ = .008) and an interaction between fixation number and ROI (*F*_(6,222)_ = 18.57, ε = .38, *p* < .001, η^2^ = .04) as well as fixation number and task (*F*_(6,222)_ = 11.44, ε = .91, *p* < .001, η^2^ = .02) implying changes from initial fixations on heads to subsequent fixations onto task-relevant areas of high saliency for color definition after the first fixation and object counting after the second fixation. This observation resulted in a significant three-way interaction for fixation number, ROI and task (*F*_(18,666)_ = 9.92, ε = .35, *p* < .001, η^2^ = .07).

To disentangle this observation with respect to the separate tasks, post hoc analyses revealed the following pattern: in the free viewing condition, participants clearly favored heads, fixating them for almost three times as long as all other ROIs and with an increasing tendency over the number of fixations (main effect of ROI: *F*_(3,111)_ = 125.58, ε = .35, *p* < .001, η^2^ = .56, main effect of fixation: *F*_(2,74)_ = 23.55, ε = .97, *p* < .001, η^2^ = .04, as well as an interaction of both factors: *F*_(6,222)_ = 24.87, ε = .37, *p* < .001, η^2^ = .17). The tasks of estimating and counting showed a slightly diminished fixation duration on heads, but displayed a similar pattern concerning the progression over fixations, particularly for heads (*estimate*: main effect of ROI: *F*_(3,111)_ = 64.41, ε = .39, *p* < .001, η^2^ = .43, main effect of fixation: *F*_(2,74)_ = 3.21, ε = 1, *p* = .047, η^2^ = .008, and interaction: *F*_(6,222)_ = 5.58, ε = .40, *p* < .001, η^2^ = .05; *count*: main effect of ROI: *F*_(3,111)_ = 35.78, ε = .37, *p* < .001, η^2^ = .26, main effect of fixation: *F*_(2,74)_ = 6.55, ε = .96, *p* = .002, η^2^ = .02 and interaction: *F*_(6,222)_ = 5.06, ε = .35, *p* < .001, η^2^ = .05). For color definition, fixations towards the vehicle (mainly included in the areas of high saliency) were quickly made from the second fixation onwards, however, the initial fixations remained directed towards the head of actors in the scene also resulting in a main effect of ROI (*F*_(3,111)_ = 41.19, ε = .43, *p* < .001, η^2^ = .22), a main effect of fixation (*F*_(2,74)_ = 10.21, ε = 1, *p* < .001, η^2^ = .03) and a significant interaction (*F*_(6,222)_ = 3.88, ε = .39, *p* = .001, η^2^ = .05). Similar to the results we found for overall fixation durations, the tasks elicit different viewing behaviors for the first fixations. That is, the more specific a task was, i.e. defining the color of the only vehicle in the picture or counting distinct blue objects, resulted in a faster drift of fixations from heads onto areas of high saliency, which largely included those features than for trials allowing broader scanning of the stimulus, i.e. to estimate the total amount of white.

### Comparison of social and non-social stimuli

When comparing the gaze behavior with regard to saliency for social and non-social stimuli, we found a significant difference in fixation density between the categories (*F*_(1,39)_ = 78.82, ε = 1, *p* < .001, η^2^ = .033) and a two-way interaction between task and stimulus category (*F*_(2,78)_ = 63.11, ε = .98, *p* < .001, η^2^ = .046) as well as ROI and stimulus category (*F*_(1,39)_ = 78.52, ε = 1, *p* < .001, η^2^ = .06) and a three-way interaction of all three factors (*F*_(2,78)_ = 62.73, ε = .98, *p* < .001, η^2^ = .12) confirming that that social scenes experienced longer fixation durations onto areas of high saliency for specific tasks like color definition and object counting, while non-social scenes experienced diminished fixations on areas of high saliency, specifically for the color definition task (Fig 4). Furthermore, a main effect of ROI (*F*_(1,39)_ = 1537.84, ε = 1, *p* < .001, η^2^ = .88) was significant, showing a preference for fixating areas of high saliency compared to those of low saliency throughout all tasks and stimuli, a main effect of task (*F*_(2,78)_ = 3.23, ε = .98, *p* = .047, η^2^ = .005) and an interaction effect between task and ROI (*F*_(2,78)_ = 3.18, ε = .98, *p* = .047, η^2^ = .01) depicting different fixation durations on areas of high saliency for different tasks across stimulus categories. With regard to task-relevant as opposed to task-irrelevant viewing behavior, we found a main effect of time (*F*_(1,39)_ = 7.91, ε = 1, *p* = .008, η^2^ = .006). Further, an interaction between task and stimulus category (*F*_(2,78)_ = 63.11, ε = 1, *p* < .001, η^2^ = .05) and between ROI and stimulus category (*F*_(1,39)_ = 78.52, ε = 1, *p* < .001, η^2^ = .09) revealed different viewing behavior on salient aspects for social and non-social stimuli with regard to different tasks, which is confirmed by a three-way interaction of task, ROI and stimulus category (*F*_(2,78)_ = 62.73, ε = 1, *p* < .001, η^2^ = .12). The interaction between stimulus category and time did not reach statistical significance (*F*_(1,39)_ = 0.75, ε = 1, *p* = .39, η^2^ = .0002). Accordingly, a ROI by stimulus category by time interaction was not significant either (*F*_(1,39)_ = 0.73, ε = 1, *p* = .39, η^2^ = .0006). Considering different viewing behavior across tasks, an interaction between task and time was still evident (*F*_(2,78)_ = 11.33, ε = .97, *p* < .001, η^2^ = .01) as well as a three-way interactions of task, ROI and time (*F*_(2,78)_ = 13.17, ε = .97, *p* < .001, η^2^ = .03) and task, stimulus category and time (*F*_(2,78)_ = 6.41, ε = 1, *p* = .003, η^2^ = .003). Furthermore, there was a two-way interaction of ROI and time (*F*_(1,39)_ = 7.88, ε = 1, *p* = .008, η^2^ = .02) and a four-way interaction of task, ROI, stimulus category and time (*F*_(2,78)_ = 6.32, ε = 1, *p* = .003, η^2^ = .01) indicating that fixations differed across tasks for both stimulus categories for low and high saliency depending on the time window.

Analyses of the first three fixations after stimulus onset revealed a main effect of ROI (*F*_(1,38)_ = 2710.73, ε = 1, *p* < .001, η^2^ = .82) clearly showing a preference for attending areas of high saliency compared to areas of low saliency, a main effect of task (*F*_(3,114)_ = 12.49, ε = .97, *p* < .001, η^2^ = .008) that showed slight changes in viewing behavior across tasks and a significant interaction between the two (*F*_(3,114)_ = 12.49, ε = .97, *p* < .001, η^2^ = .02). An additional main effect of fixation number (*F*_(2,76)_ = 126.96, ε = .96, *p* < .001, η^2^ = .06) and an interaction between ROI and fixation number (*F*_(2,76)_ = 127.09, ε = .96, *p* < .001, η^2^ = .14), as well as task and fixation number (*F*_(6,228)_ = 7.06, ε = .88, *p* < .001, η^2^ = .007) was found, implying changes in initial and subsequent fixations with a strong bias towards areas of high saliency. A three-way interaction between ROI, task and fixation number (*F*_(6,228)_ = 7.06, ε = .88, *p* < .001, η^2^ = .02) depicted that for specific tasks like color definition and object counting, areas of high saliency generally experienced longer fixations than tasks requiring broad scanning, which is similar to the data found for social stimuli. Comparing viewing behavior for social and non-social stimuli, we found no statistically significant main effect of stimulus category (*F*_(1,38)_ = 3.43, ε = 1, *p* = .072, η^2^ = .005) and no significant interaction between ROI and stimulus category (*F*_(1,38)_ = 4.07, ε = 1, *p* = .051, η^2^ = .007). However, an interaction between task and stimulus category (*F*_(3,114)_ = 14.47, ε = .47, *p* < .001, η^2^ = .06) was significant, probably driven by different fixation progressions for social stimuli. Specifically, the tasks color definition and counting objects, experienced almost reversed fixation patterns for areas of high saliency for non-social stimuli as compared to social ones implied by an interaction for fixation number and stimulus category (*F*_(2,76)_ = 8.52, ε = 1, *p* < .001, η^2^ = .002), which was especially the case for areas of high saliency (interaction for fixation number, stimulus category and ROI: *F*_(1,76)_ = 9.59, ε = 1, *p* < .001, η^2^ = .006). A statistically significant four-way interaction between ROI, task, stimulus category and fixation number was not found (*F*_(6,228)_ = 1.65, ε = .73, *p* = .134, η^2^ = .004).

## Discussion

This study aimed at investigating differences in viewing behavior for naturalistic stimuli with social or non-social content during implementation of top-down demands to measure the degree to which social attention may be able to override top-down processes. By utilizing tasks of different complexity, we hoped to witness a gradation of potential influence on social information processing. Indeed, we found significant differences in viewing behavior across tasks. In the free-viewing condition, participants displayed a clear preference for social aspects, especially for heads, while tasks, which required gaze towards distinct non-social aspects of a scene (e.g. defining the color of a vehicle or counting objects of the same color) most effectively drew attention away from social features, yet without ever eliminating fixations on heads. Therefore, it seems that not necessarily the difficulty, but the specificity of a task interferes with social attention to a certain extent. These results are in line with Yarbus [16] and a corresponding follow-up study by DeAngelus & Pelz [17] using seven different tasks, in which those questions addressing global or multiple features resulted in more spatially distributed patterns of fixations, whereas free-viewing and tasks requiring assessment of the social feature itself produced fixations that fell primarily on faces and figures. Yarbus concluded that the observers’ fixations reflected the most informative regions for the task at hand, which is central to the “bottom-up/top-down debate”, with the invention of saliency maps supporting mere bottom-up models. However, most of these tasks necessitated scanning of social features (e.g., “Give the ages of the people”) and therefore primary elements referring to faces and figures, were invariably fixated. The tasks used in our experiments deliberately focused on non-social aspects of a scene to investigate whether fixations indeed reflected the most informative regions. According to our results, this is not necessarily the case, as heads were preferentially attended despite task requirements. Specifically, even though visually salient image regions included task-relevant details, visual exploration towards heads remained superior to saliency in all tasks. This preference for social features, was present even though participants received the task instruction before stimulus onset and could therefore prepare by generating category-specific representations.

Additionally, the interference of social attention by top-down mechanisms was only manifested for the duration of task resolution and drastically reverted back to the pattern found in free-viewing, where social information, especially heads, regained their initial preference. This return was visible for all tasks and resulted in significant differences in favor of social aspects compared to salient areas of the scene, which agrees with findings of Birmingham, Bischof & Kingstone [15,25] implying a hierarchy of selection for social stimuli. Although heads gained the most attention, bodies represented the second most fixated regions in free-viewing and post-task gaze behavior. The more specific a task became, however, the more bodies forfeited their focus to task-relevant details. Nevertheless, these details did not influence viewing behavior after task completion such that bodies regained relevance, towering physical saliency, similar to the pattern seen for heads. For non-social stimuli however, viewing behavior did not change as drastically over different tasks or time windows and mainly revealed a preference for fixating highly salient image regions.

Generally, studies involving explicit tasks to modulate attentional orienting to social features have primarily used isolated facial stimuli in comparison to objects (e.g. [26–28]). Herein, most results confirm faster detection of faces compared to non-social objects. However, isolation of a social cue avoids other potentially important aspects driving gaze selection as is the case in the real world. Hence, complex naturalistic scenes enable more general analyses of gaze patterns towards social features and consider potentially competing factors. A study of Kuhn and colleagues [29] investigated top-down effects in the form of instructions to assess the modulation of gaze during both live presentation or that of a video depicting a magician as social feature. Herein, instructions aimed at directing attention away from the social aspect (“keep your eyes on the cards”) modulated viewing behavior in some participants, indicating that viewers have certain top-down control, yet other participants’ gaze persevered on the face despite the instruction, which implies a residual bias towards the face in competition with top-down control. These results are consistent with our findings, as we also find task effects, although the preference to attend faces remained across all tasks. Furthermore, gaze-following studies have shown that humans seem to have a strong predisposition to follow or imitate a social cue, even when this is obstructive to task performance [30,31]. Coherently, the study of Birmingham, Bischof and Kingstone [15] investigated the selection of gaze information as suggested by Yarbus in a more representative manner using multiple complex stimuli and three different task instructions. Their results substantiate our findings as such that attention was captured by eyes and heads of people in the scene regardless of the task. In non-social settings, such as outdoor scenes, however, task-demands counteracted sensory signals fully [32]. Although this top-down override appears to be rather strong in a non-social context, attention towards social aspects seems to withstand such counteraction, according to the results of Birmingham et al., as well as our own. However, the tasks chosen by Birmingham and colleagues encouraged scanning of the social aspects once again, as one goal was to investigate the influence of social content and activity in the scene on gaze behavior towards the eyes. Nevertheless, this makes our results all the more enlightening concerning the natural preference to select social aspects and the resilience of social attention when faced with behaviorally significant competition. Furthermore, attention towards salient image regions remained comparable between social and non-social scenes in the current study with a stronger focus on areas of high as opposed to those of low saliency. Hence, the results discussed above seem to mainly rely on attention processes towards the social scene aspects. We further addressed the influence of saliency within these social aspects and although results indicate that saliency does influence allocation of attention to more salient regions within head and body ROIs in a scene, the difference between high and low saliency was smaller for these social areas compared to that of the background. This is yet another indicator that social aspects experience a different viewing behavior than non-social ones.

According to Sharma [21], who compared the performance of 10 different algorithms, most saliency models are statistically close to each other in their correspondence with human eye fixations. The fact that we observed a preference to regard human beings over physically salient aspects, implies that saliency algorithms may struggle to accurately describe viewing behavior in the presence of social information. Furthermore, this raises the question about which underlying processes are involved in the direction of and prioritized attention towards social features. It is possible that some stimuli attract attention because of some form of contingency that is hard wired in the brain by learning, development or genetics [33]. Hence, some might argue that observers might have simply been interested to select faces voluntarily indicating that social attention is a special form of top-down mechanism that we have internalized through experience. However, a few studies demonstrating an early onset of preferential orienting towards social information argue against this notion. For example, Fletcher-Watson and colleagues [14] did not only find a bias towards looking at a social stimulus compared to a non-social one during free-viewing, but also reported that this bias was already evident in the first fixation occurring as early as 100ms after stimulus presentation. Another example is that of Crouzet, Kirchner & Thorpe [34] who showed that saccades towards faces occur as early as 100-110ms, even in competition with simultaneously presented target stimuli, suggesting that this mechanism is not completely under instructional control. Also our own analyses are in accord with this, as the first fixations reveal an initial, possibly almost reflexive bias to attentionally select social information in a complex scene. According to our results, top-down goals of for example detecting a vehicle, seem to affect the second fixation at the earliest, however, the first fixations are primarily directed at heads. Similarly, electrophysiological [35] and imaging studies [36] found faces to be categorized as early as 100ms after stimulus onset, whereas objects required around 200ms for categorization. Further support can be found in social orienting paradigms, as shifts towards gazed at locations occur rapidly, within a few hundred milliseconds after a gazing face is presented (e.g. [37]) even if eye direction is counter-predictive of target location [38]. Yet another example can be drawn from studies with infants who show looking preferences for faces, which led Johnson [39] to conclude that the adult ‘social brain’ may be developmentally founded through a subcortical face-detection system involving the superior colliculus, pulvinar and amygdala. This route was initially proposed by clinical studies concerning patients with hemispacial neglect whose visual extinction towards stimuli in their neglected field was revoked if the elements were arranged in the pattern of a face [40,41]. Also Dolan and colleagues [42] found that ambiguous pictures activated face-processing regions only when observers recognized the pictures as depicting faces. These studies indicate that there may be a separate route for face detection [39].

On the grounds of our study, we cannot conclude definitely whether mechanisms of social attention are voluntary or reflexive, nevertheless, this rationale suggests very early processing of social compared to non-social information that seem to precede top-down mechanisms. Further studies are needed to confirm this idea and differentiate between automatic shifts of attention and conscious selection of scene elements. This may be accomplished through a gaze-congruent paradigm to emphasize top-down control (e.g. [43–46]) in comparison to very short presentation times of stimuli that preclude active exploration of visual scenes (e.g., 100ms) for stressing bottom-up mechanisms (see review [47]) or dot-probe variants for stimuli comprising a social “side” versus a non-social one (e.g. [26]). On the other hand, we do not state that social attention can be categorized as a clear-cut bottom-up process either that is driven by low-level physical features, supported by our finding that physical saliency is not an accurate predictor of attentional focus. Furthermore, studies investigating the “pop-out” effect of faces have been inconclusive [48]. Instead, we suggest a potential additional attention mechanism that drives the social override. To examine the neural nature of this override, functional neuroimaging may offer insight into networks that drive social attention independently of bottom-up and top-down mechanisms. Herein, we suggest a subcortical route rapidly conveying information to the amygdala. This has previously also been suggested for fear recognition, as the amygdala responds rapidly to emotional faces, especially fearful expressions (e.g. [49]) and seems to be implicated in eliciting gaze shifts towards specific diagnostic facial features (e.g. [50,51]). Furthermore, Fitzgerald and colleagues [52] concluded from an fMRI study that the amygdala is not selective of any particular emotion category, but instead, may have a more general-purpose function in processing facial information. Therefore, the amygdala might be involved in orienting attention towards social features in general and faces in particular in natural every-day environments similar to the suggestions of Johnson [39] concerning a subcortical route for face detection and identification.

Although the current study revealed important insights into the influence of top-down demand on the extent of social attention, some limitations need to be acknowledged. First, we cannot exclude the possibility of carry-over effects in this study, as stimuli integrated blue objects or cars even for free-viewing tasks. Second, specific image regions were not entirely confined to one task (e.g. cars could have bright or bluish colors, therefore being relevant for estimation and counting tasks, respectively). Yet this further strengthens our findings as even though carry-over effects may have been present in visual exploration, social information was still preferentially selected, despite the fact that it was irrelevant and rather disadvantageous to solving the task. Third, some might argue about the implied “liveliness” of human beings in social versus non-social stimuli and we cannot fully negate this assumption with our data. However, some pictures included animals, which would have contributed to low or highly salient areas of the scene and challenging fixations towards human features. Furthermore, Crouzet, Kirchner & Thorpe [34] explicitly investigated saccade velocity towards human faces compared to animals and found that while animals can be detected as early as 120-130ms after stimulus onset, saccades to human faces are even faster with the earliest saccades occurring at 100-110ms.

## Conclusion

We have shown that while people will fixate other parts of a complex visual scene to extract relevant information, their preferential bias is to fixate faces of others. Furthermore, gaze selection seems to be driven by the goal to extract social information, even if this visual selection is irrelevant or even disadvantageous to solving a task. Our results therefore allow us to suggest that bottom-up driven saliency seems to have less influence on attentional orienting in a social context. Although top-down demands interfere with social attention, they do not extinguish fixations on conspecifics and they are strongly confined to the time of the task. This supports our hypothesis that social stimuli may engage special perceptual processing and provide exclusive access to the priority map, enabling a partial override of top-down as well as bottom-up mechanisms in attentional orienting. Future studies will have to reveal whether there is a qualitative instead of a quantitative difference between social and non-social attention and whether we have specific neural circuits that are supporting the attentional prioritization of human beings.
